# Relationship between the experience of being a bully/victim and mental health in preadolescence and adolescence: a cross-sectional study

**DOI:** 10.1186/s12991-017-0160-4

**Published:** 2017-10-18

**Authors:** Sachiko Kozasa, Arata Oiji, Akio Kiyota, Tetsuji Sawa, Soo-yung Kim

**Affiliations:** 1 Graduate School of Medical Sciences, Kitasato University, Sagamihara, Kanagawa Japan; 2 Division of Child and Adolescent Psychiatry, Department of Pediatrics, Faculty of Medicine, Oita University, Yufu, Oita Japan; 3 Division of Psychology, School of Psychology, Tokyo University of Social Welfare, Kita-Ku, Tokyo, Japan; 4 National Rehabilitation Center for Persons with Disabilities, Tokorozawa, Saitama Japan

**Keywords:** Bullying, Mental health, Adolescence, Preadolescence, Japan

## Abstract

**Background:**

Several studies have proven that the experiences of being bullied or bullying others are associated with poor mental health among adolescent youths. Our study aims to investigate the relationship between the experience of the bully/victim and mental health among preadolescents and adolescents.

**Methods:**

Subjects were the Japanese fifth and sixth grade elementary school students (preadolescents: mean age = 11.3 years; *n* = 338) and junior high school students (adolescents: mean age = 13.8 years; *n* = 486). A self-report questionnaire was administered containing items concerning the experience of being a bully/victim and the Youth Self Report (YSR).

**Results:**

Four groups relating to the experience of being a bully/victim were formed: “Victim Only,” “Bully Only,” “Victim and Bully,” and “Neither.” Approximately 65% of preadolescents and approximately 25% of adolescents engaged in bullying behaviors. Of these, the rate of participants in the “Bully Only” group was low, and that in the “Victim and Bully” group was high. Regarding the relationship between the experience of being a bully/victim and mental health, both preadolescents and adolescents of the “Victim Only” group had significantly higher scores on the YSR’s internalizing problems compared with the “Neither” group. Moreover, both preadolescents and adolescents of the “Bully Only” group had significantly higher scores on the YSR’s externalizing problems compared with the “Neither” group. Regarding the relationship between the experience of being a bully/victim and suicidal ideation for both preadolescent and adolescent girls, the relative risks of suicidal ideation were significantly higher in the “Victim and Bully” group than in the “Neither” group.

**Conclusions:**

Preadolescents indicated a higher rate of bullying behaviors than adolescents. In both preadolescents and adolescents, different effect patterns on mental health were found for the “Victim Only,” “Bully Only,” and “Victim and Bully” groups. The prevention and intervention methods for mental health should be tailored according to the type of experience associated with being a bully/victim and according to the developmental stages of preadolescence or adolescence.

## Introduction

Bullying at school is widely recognized as a major public health problem for adolescent youths. Olweus defined bullying as follows: “A student is being bullied or victimized when he or she is exposed, repeatedly and over time, to negative actions on the part of one or more other students. It is a negative action when someone intentionally inflicts injury or discomfort upon another, which is implied in the definition of aggressive behavior. Negative actions can be carried out by physical contact, by words, or in other ways, such as making faces or mean gestures, and intentional exclusion from a group” [1]. We used Olweus’s definition in this study.

Nansel et al. [2] studied young people in 25 countries and found that 5–20% had been victims, 2–3% had been bullies, and 1–20% had been both bullies and victims. Craig et al. studied samples of 11-, 13-, and 15-year-old school children in 40 countries and found that exposure to bullying varied across countries, with estimates ranging from 8.6 to 45.2% among boys and from 4.8 to 3.5% among girls [3]. Tochigi et al. [4] studied 19,436 Japanese students in 45 public junior high schools (7th–9th grade) and 28 senior high schools (10th–12th grade) and found that 15,385 (85.1%) were uninvolved in bullying; out of the remainder, 1346 (7.4%) were pure bullies, 859 (4.7%) were pure victims, and 514 (2.8%) were both bullies and victims. These studies proved that experiences of bullying and/or being bullied are common and universal but the frequencies of such experiences were different among these studies.

Among adolescents, several studies have proved that experiences of being bullied and/or bullying others are associated with poor mental health. Victims have generally been shown to have internalizing problems [5, 6] and depression [7]. Students with internalizing problems were more likely to be victims than bullies [5], and bullies were reported to have externalizing problems [5, 8]. However, Yang et al. found that depressive symptoms were common not only among victims, but also among bullies [9]. Both victims and bullies have been shown to be associated with various internalizing and externalizing behaviors [5, 6, 8, 10–13]. Furthermore, several studies have shown that incidences of being bullied and/or bullying others put adolescents at risk of suicidal ideation [10, 14–19].

It is considered that the impacts of experiences relating to bullying in preadolescence may differ from those in adolescence because the quality and significance of peer relationships are different between adolescence and preadolescence. However, few studies have investigated the relationships between the experiences of being a bully/victim in preadolescence and mental health. Particularly, very few studies have attempted to examine the relationships between the experiences of being a bully/victim and mental health in preadolescents [20]. Among the few studies, Karatas and Ozturk reported that students (11.6 ± .53 years) exposed to bullying were significantly more likely to experience headaches, feel bad, crying, restlessness, nervousness, sleeping problems, and dizziness [21]. Williford et al. [22] reported that elementary school (10.2 years) bullies with higher levels of depressive symptoms were less likely than other students to move to an uninvolved status in the first year of middle school. They noted the implications for school-based prevention programs during the move to middle school. However, little is known about the association between the types of experiences of being a bully/victim and mental health in preadolescence. The authors think that it is significant to conduct research on the relationship between mental health and the experiences of being a bully/victim in preadolescence as well as adolescence.

This study aims to examine whether and how the experiences of being a bully/victim in preadolescence were associated with mental health, including internalizing and externalizing problems, and to compare the results with those in adolescents. Many studies indicated that the associations between bullying and mental health or risk of suicidal ideation differ by gender among adolescents [8, 15–17, 23–25]. The present study examined associations of mental health with being bullied or bullying separately for boys and girls.

## Subjects and methods

### Subjects

We approached the principals of six elementary and junior high schools in three areas of western Tokyo and asked them to participate in this study. We provided them with both written and verbal explanations about what the study involved. To execute this study, we distributed written explanations of this study to the teaching staff at the schools in which we had obtained the principal’s approval. We obtained approval to conduct the study from the principal and teaching staff at three schools. Requests to take the survey were made to the students in two mid-sized (from 500 to 700 students) public elementary schools and one public junior high school. The surveys were performed at each school in December 2014. Subjects were the fifth and sixth graders in the elementary schools, and the seventh, eighth, and ninth graders in the junior high school. Written explanations of what the study involved were distributed to the children’s parents/guardians. These explanations included a form for opting out of the study, so that those not wishing for their child to participate could submit the signed opt-out form to the school. The surveys were self-administered questionnaires to be completed anonymously in each class during times for extracurricular activities. The surveys were inserted into individual envelopes and handed out to all the students. The students were asked to write their grade, class, and their class identification number on the envelope (in Japan, every student in a class is assigned a number based on their position in the class roster). The cover page of the survey explained the survey’s purpose and methodology. It also explained that it was fine to leave any or all the answers blank and that there was no penalty for doing so. Before the students started the survey, we had the teacher verbally reiterate these points. The first item of the actual survey was to answer the question: “Are you willing to participate in this study?” Consent to participate was considered obtained if the student marked: “I am willing to participate.” After the students completed the surveys, each school collected the surveys and mailed them to the Developmental Psychiatry Laboratory of the Graduate School of Medical Science, Kitasato University. The schools discarded the envelopes of students whose parents/guardians opted out of the study or of those who had marked: “I am not willing to participate.”

### Measures

#### Questionnaire items concerning bullying at school

A preliminary survey was administered to the fifth and sixth graders at an elementary school, and the seventh, eighth, and ninth graders at a junior high school in west Tokyo, in order to develop questions concerning bullying for the actual study. Teachers explained the purpose of this survey and obtained consent from the children and their parents/guardians. This preliminary survey asked whether the student had ever been a victim or a perpetrator of bullying at school. Those who had been involved in bullying were asked to describe the experience in their own words. Researchers examined these responses and divided the content into six categories: “were hit or kicked by someone/engaged in hitting or kicking someone”; “were ignored or left out by someone/ignored or left someone out”; “were teased or had bad things said or written about themselves/teased someone or said or wrote bad things about someone”; “had malicious videos or photos of themselves posted on the Internet or things written about themselves on the Internet they did not like/wrote malicious things about or posted videos or photos of someone on the Internet”; “had their possessions or money stolen, damaged, or hidden/stole, damaged, or hid someone’s possessions or money”; and “were threatened or forced to do something they did not want to do/threatened or forced someone to do something they did not want to do.” We named these six types of bullying; “physical abuse”; “ignoring/leaving someone out”; “verbal abuse”; “cyber-bullying”; “stealing/hiding/damaging someone’s possessions”; and “threatening someone.”

The survey explored if the student had been involved in any of these six types of bullying at school as a victim or perpetrator since the beginning of the school year.

#### The Youth Self Report (YSR) [26]

The Achenbach approach to emotional and behavioral assessment offers a dimensional conceptualization of child psychiatric problems covering a wide range of symptom domains. The YSR is a questionnaire, designed to be completed by adolescents aged 11–18 years, and consists of 112 problem items covering different symptoms/behaviors, each to be rated on a three-point scale. All ratings refer to symptoms or problems experienced during the preceding 6 months. The YSR can be scored on the total problem scale, which is the sum of the scores for each problem item, and the following eight syndrome scales: withdrawn, somatic complaints, and anxious/depressed (together comprising the broadband internalizing scale); social problems; thought problems; and attention problems (which are not part of either the internalizing or externalizing scales); and delinquent behavior and aggressive behavior (together comprising the externalizing scale). The Japanese version of this tool was translated and standardized by Kuramoto et al. [27].

### Statistical analysis

The survey results were analyzed using the SPSS Statistics Base and SPSS Missing Values in IBM SPSS Statistics 23. Because 36.6% of the subjects left one or more of the 112 YSR questions unanswered, missing values in the YSR were imputed using multiple imputations. Experiences of bully/victim were categorized into four groups: “Victim only” (“Victim”), “Bully only” (“Bully”), “both Bully and Victim” (“Bully + Victim”), and “Neither.” Individuals who experienced even one of the six types of bullying (physical abuse, ignoring/leaving someone out, verbal abuse, cyber-bullying, stealing/hiding/damaging someone’s possessions, and threatening someone) as a victim but not as a bully were classified as “Victim” Those with no experience as a victim but who engaged in one or more types of bullying were classified as “Bully.” Those who had experienced one or more types as a bully and/or a victim were classified as “Bully + Victim.” Finally, those with no experience of being either a victim or bully were classified in the “Neither” category.

To test for differences among these four groups in terms of their means on the YSR subscale and dimension scores, one-way analyses of variance (one-way ANOVAs) were performed separately for elementary school boys, elementary school girls, junior high school boys, and junior high school girls. Post hoc tests were performed using the Tukey’s test. The significance threshold used was set at *p* < .01.

In addition, we calculated the relative risk of suicidal ideation between the bullying experience groups. Individuals were judged to have had suicidal ideation if they responded one or two on the YSR items: “I deliberately tried to hurt or kill myself” and “I think about killing myself,” where 0 = “not true of me,” 1 = “somewhat true of me,” and 2 = “very true of me.” The total score for the two responses was used to categorize individuals according to whether they had engaged in suicidal ideation. Individuals with a total score of 0 were assigned to the “No Suicidal Ideation” group and those with a score of 1–4 were assigned to the “Suicidal Ideation” group. The relative risk of suicidal ideation was calculated for the three groups: “Victim,” “Bully,” and “Bully + Victim,” while the “Neither” group was used for comparison.

## Results

### Subjects

The surveys were performed at each school in December 2014. Because some students were absent on the day the survey was performed, 925 of the eligible students took the survey. Of these, the parents of 22 students opted out of the study, 31 students declined to participate, 14 did not answer the consent question, and 31 students were excluded because one or more of the answers to the demographic questions about grade, age, and gender were missing. Thus, the analysis was performed for 827 subjects.

These subjects consisted of 338 fifth and sixth grade elementary school students, and 489 seventh, eighth, and ninth grade junior high school students. The elementary school (preadolescent) sample consisted of 175 boys and 163 girls, while the junior high school (adolescent) sample consisted of 243 boys and 249 girls. The mean age for preadolescent sample was 11.26 years (SD = .67) and the mean age for the adolescence sample was 13.76 years (SD = .95).

### Experiences of being a bully/victim

Among both preadolescent and adolescent subjects, regardless of gender, the percentage of students in the Bully group were the lowest, while those in the Bully + Victim group were much higher (Table 1).

**Table 1:** Bullies and/or victims among girls and boys

	Neither, *n* (%)		Victim, *n* (%)		Bully, *n* (%)		Bully + Victim, *n* (%)		Total, *n*	
	Boys	Girls	Boys	Girls	Boys	Girls	Boys	Girls	Boys	Girls
Preadolescents	44 (26.2)	69 (42.6)	35 (20.8)	52 (32.1)	13 (7.7)	9 (5.6)	76 (45.2)	32 (19.8)	168	162
Adolescents	147 (62.6)	154 (63.6)	30 (12.8)	38 (15.7)	23 (9.8)	9 (3.7)	35 (14.9)	41 (16.9)	235	242

The numbers do not include individuals who did not answer one or more of the demographic questions (16 preadolescents and 8 adolescents)

### YSR dimension and subscale scores and experiences of bully/victim

One-way ANOVAs and Tukey’s tests were used to examine if there were significant differences among the four bullying experience groups in terms of the mean values for the ten YSR subscale and dimension scores.

Results for preadolescent boys showed that the Bully + Victim group had significantly higher mean scores than those for the Neither group on withdrawn, social problems, delinquent behavior, aggressive behavior, and the externalizing scale. In addition, the mean score for the externalizing scale for the Bully + Victim group was significantly higher than that for the Victim group. Compared with the mean scores of the Neither group, those for the Victim group were significantly higher for the withdrawn and social problem subscales, and the mean scores for the Bully group were significantly higher for the aggressive behavior subscale (Table 2).

**Table 2:** Scores of the Youth Self Report by experiences of being a bully/victim (preadolescent boys)

Scale	Neither, *n* = 44		Victim, *n* = 35		Bully, *n* = 13		Bully + Victim, *n* = 76		*F* value	*p* value	Post hoc (Tukey)
	Mean	SD	Mean	SD	Mean	SD	Mean	SD			
Withdrawn	.87	1.483	2.74	2.631	1.77	2.351	2.67	2.637	5.304	.0016*	*V*, *B*/*V* > *N*
Somatic complaints	1.57	2.350	2.29	2.388	2.20	3.118	2.36	2.588	1.353	.2598	Not significant
Anxious/depressed	3.66	4.291	6.91	5.732	5.54	5.251	6.97	5.774	2.940	.0353	Not significant
Social problems	2.45	2.524	4.62	3.128	3.75	1.974	4.20	2.923	4.290	.0062*	*V* > *N*
Thought problems	1.43	1.408	2.43	2.634	1.94	1.322	2.46	2.154	2.158	.0953	Not significant
Attentions problems	4.71	3.503	5.78	3.590	6.21	3.830	6.11	3.556	1.406	.2433	Not significant
Delinquent behavior	1.14	1.212	1.45	1.562	2.44	1.687	2.81	2.996	7.444	.0001**	*B*/*V* > *N*
Aggressive behavior	4.07	3.144	6.04	4.390	8.86	5.253	9.38	5.588	10.300	.0000***	*B*/*V*, *B* > *N*
Internalizing scale	6.06	6.621	11.72	8.502	9.35	10.036	11.78	8.924	2.694	.0489	Not significant
Externalizing scale	5.21	4.043	7.48	5.658	11.30	6.542	12.19	7.928	10.516	.0000***	*B*/*V* > *V*, *N*

Multiple imputation was used to impute missing values. One-way ANOVAs were performed. Comparisons performed using the Turkey’s test

N, Neither; B, Bully; V, Victim; B/V, Bully + Victim

* *p* < .01, ** *p* < .001, *** *p* < .0001

For preadolescent girls, the mean scores for the Bully + Victim group were significantly higher than those for the Neither group for somatic complaints, anxious/depressed, social problems, thought problems, delinquent behavior, aggressive behavior, and for the internalizing and externalizing scales. The mean scores for the Victim group were significantly higher than those for the Neither group for anxious/depressed, social problems, and attention problems. The mean scores for the Bully group were significantly higher than those for the Neither group for aggressive behavior and the externalizing scale (Table 3).

**Table 3:** Scores of the Youth Self Report by experiences of being a bully/victim (preadolescent girls)

Scale	Neither, *n* = 69		Victim, *n* = 52		Bully, *n* = 9		Bully + Victim, *n* = 32		*F* value	*p* value	Post hoc (Tukey)
	Mean	SD	Mean	SD	Mean	SD	Mean	SD			
Withdrawn	2.05	1.967	2.83	2.728	2.10	1.455	3.62	2.468	3.139	.0273	Not significant
Somatic complaints	1.79	2.763	3.64	3.116	.96	1.216	3.54	2.663	5.623	.0012*	*B*/*V* > *N*
Anxious/depressed	3.76	3.294	7.67	5.964	4.93	3.361	8.61	5.151	10.193	.0000***	*B*/*V*, *V* > *N*
Social problems	2.76	1.812	4.92	2.889	3.92	2.151	4.68	2.968	6.938	.0000***	*V*, *B*/*V* > *N*
Thought problems	1.44	2.090	2.40	2.310	2.67	2.495	2.88	2.366	4.833	.0031*	*B*/*V* > *N*
Attention problems	4.11	2.647	6.50	3.235	5.82	3.942	6.18	3.076	5.440	.0014*	*V* > *N*
Delinquent behavior	1.02	1.281	1.65	1.744	2.16	1.938	2.60	2.276	8.691	.0000***	*B*/*V* > *V*, *N*
Aggressive behavior	4.21	3.744	6.61	4.244	10.79	5.577	9.31	4.703	13.394	.0000***	*B*, *B*/*V* > *N*
Internalizing scale	7.51	6.400	13.79	9.848	7.85	4.992	15.30	7.635	8.210	.0000***	*B*/*V* > *N*
Externalizing scale	5.23	4.589	8.26	5.210	12.95	7.085	11.91	6.549	12.971	.0000***	*B*, *B*/*V* > *N*, *B*/*V* > *V*

Multiple imputation was used to impute missing values. One-way ANOVAs were performed. Comparisons performed using the Turkey’s test

N, Neither; B, Bully; V, Victim; B/V, Bully + Victim

* *p* < .01, ** *p* < .001, *** *p* < .0001

For adolescent boys, mean scores were significantly higher in the Bully + Victim group compared with the Neither group in terms of social problems, attention problems, aggressive behavior, and the externalizing scale. Mean scores for the Victim group were significantly higher than those for the Neither group for withdrawn, anxious/depressed, social problems, attention problems, and the internalizing scales. In addition, the mean score for the Victim group for the social problem subscale was significantly higher than that for the Bully group. The Bully group exhibited a significantly higher mean score than did the Neither group for attention problems and aggressive behavior (Table 4).

**Table 4:** Score of the Youth Self Report by experiences of being a bully/victim (adolescent boys)

Scale	Neither, n = 146		Victim, *n* = 30		Bully, *n* = 23		Bully + Victim, *n* = 35		*F* value	*p* value	Post hoc (Tukey)
	Mean	SD	Mean	SD	Mean	SD	Mean	SD			
Withdrawn	1.98	2.206	3.95	3.422	2.57	2.537	2.80	1.997	6.538	.0003**	*V* > *N*
Somatic complaints	1.68	2.972	2.20	2.491	3.18	3.822	2.27	2.339	1.972	.1192	Not significant
Anxious/depressed	4.67	4.757	9.32	7.161	6.34	5.916	6.62	4.479	6.815	.0002**	*V* > *N*
Social problems	2.75	2.252	5.59	2.921	3.13	2.096	5.06	2.807	15.998	.0000***	*V*, *B*/*V* > *N*; *V* > *B*
Thought problems	1.93	2.292	2.27	2.363	2.39	2.911	3.03	2.695	2.021	.1119	Not significant
Attention problems	4.80	3.110	7.37	2.977	6.96	2.529	7.29	2.442	12.146	.0000***	*V*, *B*/*V*, *B* > *N*
Delinquent behavior	1.72	2.110	2.13	1.961	3.05	2.174	2.43	1.481	3.160	.0255	Not significant
Aggressive behavior	5.68	4.296	7.30	5.691	9.97	5.251	9.80	5.389	10.626	.0000***	*B*, *B*/*V* > *N*
Internalizing scale	8.19	8.637	14.90	11.013	11.87	11.053	11.51	6.298	4.711	.0034*	*V* > *N*
Externalizing scale	7.40	5.807	9.44	7.157	13.03	6.459	12.23	6.197	8.764	.0000***	*B*/*V* > *N*

Multiple imputation was used to impute missing values. One-way ANOVAs were performed

N, Neither; B, Bully; V, Victim; B/V, Bully + Victim

* *p* < .01, ** *p* < .001, *** *p* < .0001

For adolescent girls, the mean scores for the Bully + Victim group were significantly higher than those for the Neither group for all YSR dimensions and subscales. In addition, the mean scores for the Bully + Victim group were significantly higher than those for the Victim group for delinquent behavior and the externalizing scale. Additionally, the mean scores for the Bully + Victim group were significantly higher than those for the Neither group on both internalizing and externalizing problems. The mean scores for the Victim group were significantly higher than those for the Neither group for withdrawn, somatic complaints, anxious/depressed, social problems, and the internalizing scale. Finally, the mean score for the Bully group was significantly higher than that for the Neither group for delinquent behavior (Table 5).

**Table 5:** Score of the Youth Self Report by experiences of being a bully/victim (adolescent girls)

Scale	Neither, *n* = 154		Victim, *n* = 38		Bully, *n* = 9		Bully + Victim, *n* = 41		*F* value	*p* value	Post hoc (Tukey)
	Mean	SD	Mean	SD	Mean	SD	Mean	SD			
Withdrawn	2.22	2.151	4.21	2.951	3.11	2.147	3.67	2.654	9.370	.0000***	*V*, *B*/*V* > *N*
Somatic complaints	2.10	2.627	4.08	2.794	3.82	3.777	4.14	3.670	10.501	.0000***	*B*/*V*, *V* > *N*
Anxious/depressed	5.62	4.917	10.86	6.561	8.22	5.544	10.45	6.679	14.725	.0000***	*V*, *B*/*V* > *N*
Social problems	3.78	2.519	5.76	2.476	4.85	2.425	5.32	2.631	8.792	.0000***	*V*, *B*/*V* > *N*
Thought problems	1.71	2.021	2.48	2.137	3.11	2.522	3.93	3.297	10.311	.0000***	*V*/*B* > *N*
Attention problems	6.13	3.176	7.76	2.972	7.96	3.411	8.10	3.897	5.640	.0001**	*B*/*V* > *N*
Delinquent behavior	1.34	1.559	1.95	1.707	3.56	2.404	3.71	2.452	20.704	.0000***	*B*/*V*, *B* > *N*; *B*/*V* > *V*
Aggressive behavior	6.19	4.660	8.93	5.037	11.80	5.982	12.39	6.062	17.832	.0000***	*B*/*V* > *N*
Internalizing scale	9.77	7.902	18.78	10.253	14.81	9.103	17.72	10.777	15.827	.0000***	*V*, *B*/*V* > *N*
Externalizing scale	7.54	5.794	10.88	5.931	15.36	7.506	16.10	7.599	21.477	.0000***	*B*/*V* > *V*, *N*

Multiple imputation was used to impute missing values. One-way ANOVAs were performed. Comparisons performed using the Turkey’s test

N, Neither; B, Bully; V, Victim; B/V, Bully + Victim

* *p* < .01, ** *p* < .001, *** *p* < .0001

### Experiences of being a bully/victim and suicidal ideation

For preadolescent boys, the relative risk of suicidal ideation for the Bully + Victim group was higher than that for the Neither group, with an odds ratio of 4.85 (95% CI 1.35–17.44). For preadolescent girls, the relative risk was higher for both the Bully + Victim group, with an odds ratio of 18.82 (95% CI 4.88–72.61), and for the Victim group, with an odds ratio of 9.75 (95% CI 2.66–35.79), than that for the Neither group (Table 6).

**Table 6:** Relative risk of suicidal ideation in boys and girls by experiences of being a bully/victim (preadolescents)

Suicidal ideation	Boys, *n* = 168				Girls, *n* = 162			
	Neither, *n* = 44	Victim, *n* = 35	Bully, *n* = 13	Bully + Victim, *n* = 76	Neither, *n* = 69	Victim, *n* = 52	Bully, *n* = 9	Bully + Victim, *n* = 32
	*n* (%)	*n* (%)	*n* (%)	*n* (%)	*n* (%)	*n* (%)	*n* (%)	*n* (%)
No	40 (93.0)	26 (76.5)	11 (84.6)	55 (73.3)	64 (95.5%)	35 (68.6)	7 (87.5)	17 (53.1)
Yes	3 (7.0)	8 (23.5)	2 (15.4)	20 (26.7)	3 (4.5)	16 (31.4)	1 (12.5)	15 (46.9)
OR	1.00	4.10*	2.42	4.85*	1.00	9.75*	3.05	18.82*
95% CI		1.00–16.90	.36–16.36	1.35–17.44		2.66–35.79	.28–33.39	4.88–72.61

* Indicates a significant difference in the relative risk of suicidal ideation

For adolescent boys, compared with the Neither group, the relative risk of suicidal ideation for the Victim group was higher, with an odds ratio of 4.28 (95% CI 1.70–10.77). For adolescent girls, the relative risk was higher for both the Victim group, with an odds ratio of 3.92 (95% CI 1.82–8.44), and the Bully + Victim group, with an odds ratio of 2.51 (95% CI 1.16–5.43), than that for the Neither group (Table 7).

**Table 7:** Relative risk of suicidal ideation in boys and girls by experiences of being a bully/victim (adolescents)

Suicidal ideation	Boys, *n* = 235				Girls, *n* = 242			
	Neither, *n* = 146	Victim, *n* = 30	Bully, *n* = 23	Bully + Victim, *n* = 35	Neither, *n* = 154	Victim, *n* = 38	Bully, *n* = 9	Bully + Victim, *n* = 41
	*n* (%)	*n* (%)	*n* (%)	*n* (%)	*n* (%)	*n* (%)	*n* (%)	*n* (%)
No	130 (89.0)	19 (65.5)	18 (81.8)	28 (80.0)	126 (82.9)	21 (55.3)	6 (66.7)	27 (65.9)
Yes	16 (11.0)	10 (34.5)	4 (18.2)	7 (20.0)	26 (17.1)	17 (44.7)	3 (33.3)	14 (43.1)
OR	1.00	4.28*	1.81	2.03	1.00	3.92*	2.42	2.51*
95% CI		1.70–10.77	.54–6.00	.76–5.40		1.82–8.44	.56–1.28	1.16–5.43

The figures do not include one boy who did not respond to either of the Youth Self Report question items: “I deliberately try to hurt or kill myself” or “I think about killing myself.”

* Indicates a significant difference in the relative risk of suicidal ideation

## Discussion

### Experiences of being a bully/victim at school

In total, 73.8% of preadolescent boys, 56.4% of preadolescent girls, 37.4% of adolescent boys, and 36.4% of adolescent girls were involved in bullying either as a victim, a bully, or both bully and victim. Our results showed that the rate of preadolescents who had been involved in bullying was higher than that for adolescents. Rothon et al. [7] reported that those in the older age group were less likely to have been bullied in a study in which a self-administered questionnaire was completed by 11–12-year olds and 13–14-year olds. Our results supported these findings. In the present study, among the four groups, the rates of the Bully group were low and those of the Bully + Victim group were high for preadolescents and adolescents. Isolan et al. [13] also reported that the rate of those who were both bullies and victims was higher. However, in contrast to our results, several other studies have reported lower incidence of the Bully + Victim group [5, 9, 23, 28]. In the present study, the survey questions concerned six concrete behaviors that are thought to constitute bullying rather than using the word “bullying” in the survey itself. It is conceivable that the percentages of students in the Bully + Victim group were higher as a result of this methodological difference. In any case, it was revealed that it is possible for individuals in the same social group to be a bully and a victim. Possibly, Japanese preadolescent and adolescents switch between these roles. If that is the case, further study would be necessary to look at whether this may be the result of Japanese sociocultural influences.

### Relationships between experiences of being a bully/victim and mental health

This study results revealed higher scores for some of the YSR subscales for the Victim, Bully, and Bully + Victim groups compared to those in the Neither group, suggesting that the experience of being a bully/victim are associated with mental health for preadolescents and adolescents. However, the three groups differed in terms of which YSR scores were higher, that is, there were differences in the effect patterns in each group, and those patterns differed by gender for preadolescents and adolescents.

For the adolescent boys and girls, the Victim group had more internalizing problems, which support the findings of Kelly et al. [5] and Lemstra et al. [29]. For the Victim group, the mean scores for preadolescent boys for withdrawn and social problems were higher than those for the Neither group; for preadolescent girls, the mean scores for anxious/depressed, social problems, and attention problems were higher. These results may indicate that subjects who had experienced being only victims had more internalizing problems among preadolescents similar to adolescents.

For the Bully groups among preadolescents and adolescents, the mean scores were significantly higher than those for the Neither group for aggressive behavior and the externalizing scale, It is suggested that they had more externalizing problems. Our results supported those of Kelly et al. [5] and Ivarsson et al. who also found a tendency for individuals who were only bullies to have more externalizing problems [8]. Individuals who are only perpetrators of bullying may more frequently engage in aggressive and/or delinquent behavior for preadolescents and adolescents.

For the Bully + Victim groups among preadolescents and adolescents, mean scores were significantly higher than those for the Neither group for both preadolescent boys and girls for anxious/depressed, social problems, delinquent behavior, aggressive behavior, and the internalizing and externalizing scales. Mean scores for the adolescent boys were significantly higher for social problems, attention problems, aggressive behavior, and the externalizing scale. Mean scores for the adolescent girls were higher for all of the YSR dimensions and subscales. These results can be interpreted as indicating that, for the Bully + Victim group, both internalizing and externalizing problems were significantly more frequent for preadolescents and adolescents. Many studies for adolescents have shown that those who are both bullies and victims have more mental health problems than do victims or bullies alone [5, 6, 8, 10–13, 30], and our results for preadolescents and adolescents supported those findings. Among both preadolescents and adolescents, the Bully + Victim group’s scores for both the internalizing problems of victims and the externalizing problems of bullies were high, suggesting that they may have more frequent mental health problems related to both internalizing and externalizing behaviors. In addition, preadolescent girls in the Bully + Victim group scored high on all subscales, suggesting that they suffer from emotional problems in various areas. Further research is needed to investigate the reason for this.

For the Victim and Bully + Victim groups among preadolescents and adolescents, the boys and girls differed in terms of their somatic complaint scores. While there were no significant differences in the mean somatic complaint scores between the Neither group and the Victim or Bully + Victim groups for preadolescent and adolescent boys, there were significant differences in these scores for preadolescent girls between the Bully + Victim and Neither groups and for adolescent girls between the Neither group and both the Victim and Bully + Victim groups. Both preadolescent and adolescent girls who had been bullied may have been more susceptible to developing physical symptoms such as stomach pain, headaches, or fatigue. It may be characteristic for girls to develop intense somatic symptoms from internalizing the problems of being bullied even when they may also have engaged in bullying. When girls develop physical symptoms, care should be taken to consider the possibility that they are being bullied.

A better understanding of the relationship between bullying and somatic symptoms could potentially contribute to the early detection of bullying, but further analysis of this relationship is necessary.

Regarding the relationship between bullying and suicidal ideation, the results showed that for the preadolescent and adolescent boys and girls, the risk of suicidal ideation in the Victim group was significantly higher compared with the Neither group. Further, for preadolescent boys and girls and adolescent girls, the risk of suicidal ideation in the Bully + Victim group was significantly higher compared with the Neither group. This suggests the possibility that having been bullied increased the risk of suicidal ideation regardless of whether the individuals may have also been perpetrators for preadolescents and adolescents. These results supported the findings of Geoffroy et al. [25] and Kaminsky et al. [16]. Espelage et al. [24] and Luukkonen et al. [19] reported that girls who were both bullies and victims were potentially at a higher risk for suicidal ideation. Preadolescent and adolescent girls exhibited a strong association between bullying behavior and mental health.

It was found that the experiences of being a bully/victim and mental health problems were related among preadolescents. For preadolescents and adolescents, the relationship between the experiences of being a bully/victim and mental health problems was almost the same. It has been suggested that girls who have experiences of being a bully/victim are more likely to experience mental health problems than boys who have experiences of being a bully/victim.

### Study limitations and further issues

This study was a small-scale study of only three schools in one region, and the results may have some bias. Therefore, there are some limitations in extending the study results to all areas of Japan. In the future, comparative studies including more regions of Japan and larger sample sizes are needed.

This survey was conducted in each class. Therefore, to prevent social desirability bias, the survey sheet was anonymous and was collected in an envelope. However, it is possible that the students might have answered in a socially desirable direction.

It is conceivable that in studies of bullying, differences arise in the questions asked and how they are answered because of differences in how bullying is defined, how types of abuse are described, etc. This study surveyed the experiences of mental and physical abuse without using the term “bullying.” Thus, it is difficult to be certain that the same phenomenon was studied as in previous bullying studies, which may pose problems in comparing the present findings with those reported by previous studies.

In addition, while it has been reported that bullying has lasting psychological effects, in Japan, there have been no longitudinal studies. Indeed, longitudinal research on the relationship between bullying experiences and their impact on mental health is necessary.

## Conclusion

In this study, we categorized the experiences of bully/victim among a sample of the Japanese fifth and sixth grades elementary school students, and the seventh, eighth, and ninth grade junior high school students into four groups: Victim, Bully, Bully + Victim, and Neither. We examined the association between the experiences of being a bully/victim and mental health problems to identify the differences among the three types of experiences of being a bully/victim (Victim, Bully, and Bully + Victim).

Approximately 65% of preadolescents and 37% of adolescents were involved as victims, bullies, or both bullies and victims. It is notable that the preadolescents had more frequent experiences of being a bully/victim than the adolescents. Among preadolescents and adolescents, a higher number of students comprised both bullies and victims, while the number of those who were only bullies was low. This suggested that most perpetrators of bullying were also victims, while a small number were bullies only.

Both for preadolescents and adolescents, the three types of experiences of being a bully/victim were shown to be associated differently with mental health. This suggested that the care provided to individuals needs to take into consideration the different patterns of mental health problems corresponding to each type of experiences of being a bully/victim from preadolescents. In other words, prevention and intervention methods in the case of being a bully/victim should be tailored according to the type of experience relating to being a bully/victim and according to the developmental stages of preadolescence or adolescence.

This study is thought to be significant in that it adds to the little research available that examines the relationship between the experiences of being a bully/victim and mental health in preadolescents in comparison to that of adolescents.

## Abbreviation

YSR: the Youth Self Report
